# The interaction between gut microbiota and hibernation in mammals

**DOI:** 10.3389/fmicb.2024.1433675

**Published:** 2024-09-11

**Authors:** Peng Gao, Wenjing Shen, Tingbei Bo

**Affiliations:** School of Grassland Science, Beijing Forestry University, Beijing, China

**Keywords:** gut microbiota, hibernation, metabolite, immune, SCFAs

## Abstract

Hibernation, an evolved survival trait among animals, enables them to endure frigid temperatures and food scarcity during the winter months, and it is a widespread phenomenon observed in mammals. The gut microbiota, a crucial component of animal nutrition and health, exhibits particularly dynamic interactions in hibernating mammals. This manuscript comprehensively evaluates the impacts of fasting, hypothermia, and hypometabolism on the gut microbiota of hibernating mammals. It suggests that alterations in the gut microbiota may contribute significantly to the maintenance of energy metabolism and intestinal immune function during hibernation, mediated by their metabolites. By delving into these intricacies, we can gain a deeper understanding of how hibernating mammals adapt to their environments and the consequences of dietary modifications on the symbiotic relationship between the gut microbiota and the host. Additionally, this knowledge can inform our comprehension of the protective mechanisms underlying long-term fasting in non-hibernating species, including humans, providing valuable insights into nutritional strategies and health maintenance.

## Graphical abstract

During hibernation, there is an interaction between the gut microbiota and the host. The yellow part: Hibernation alters the composition of microbial communities. Due to fasting, bile and mucus from the epithelial cells of the digestive tract can serve as energy sources for some gut microbiota, leading to an increase in bacteria such as *Akkermansia muciniphila* and a decrease in bacteria such as Firmicutes. The blue part: Changes in gut microbiota may contribute to the maintenance of energy metabolism and intestinal immune function during hibernation through their metabolites. 1. The metabolic product SCFAs of gut microbiota will serve as the main energy substance. 2. The gut microbiota hydrolyzes urea in the intestine to produce ammonia, which can further synthesize amino acids and help produce new proteins for the host to utilize. 3. During hibernation, the expression of TLR5 in the intestine increases, which can minimize inflammation.
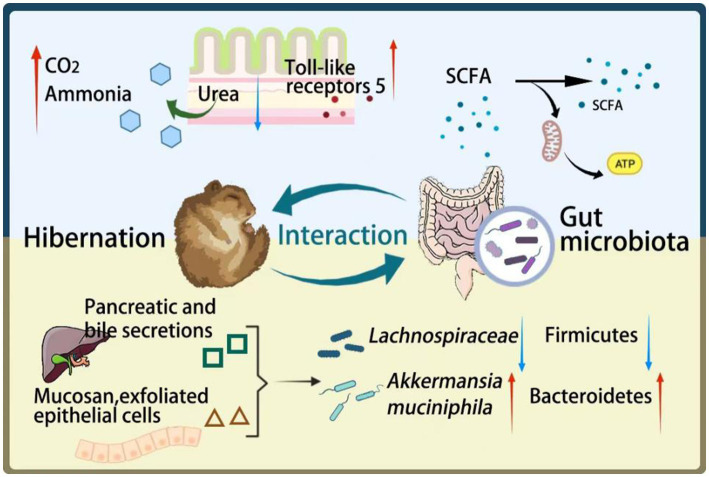

## 1 Introduction

Birds and mammals have evolved the capacity to maintain a consistently high body temperature, which allows them to perform essential survival activities independently of external temperature fluctuations (McNab, 2002). However, this physiological trait necessitates a substantial energy expenditure. To cope with this energetic demand, certain small mammals have adopted a unique physiological strategy called torpor. Torpor represents a temporary heterothermia, characterized by a reduction in body temperature, metabolic rate, and physiological function, occurring periodically within a day or during specific seasons as a response to ecological stressors (Lyman et al., 1982; Heldmaier et al., 2004; Dausmann et al., 2009; Geiser, 2013). This physiological adaptation is widespread among mammals and has also been observed in several bird species (Shankar et al., 2023; Heldmaier et al., 2004). Statistics indicate that 11 out of 25 mammalian orders exhibit torpor, highlighting its prevalence in vertebrate physiology (Heldmaier et al., 2004). Torpor manifests in two primary forms: daily torpor and hibernation. Daily torpor occurs without obvious seasonal patterns, typically lasting less than 24 h. During this period, the animal’s body temperature drops moderately, generally remaining above 20°C, and its metabolic rate during torpor is approximately 30% of its basal metabolic rate (BMR). In contrast, hibernation represents a prolonged torpor episode that occurs seasonally, often during winter. During hibernation, the animal’s body temperature can drop significantly, approaching ambient temperatures. For instance, the Arctic Ground Squirrel (*Urocitellus parryii*) has been recorded to reach a body temperature of −2.9°C during deep hibernation (Barnes, 1989). Metabolic rates during hibernation can decrease to as little as 5% of BMR, and hibernation bouts can last from several days to weeks (Heldmaier et al., 2004; Geiser, 2013; Ruf and Geiser, 2015). Notably, hibernation is not a continuous state; animals periodically awaken, dividing the hibernation period into distinct bouts (Geiser, 2013).

The significance of torpor in mammalian survival is multifaceted. It conserves energy, enabling animals to cope with the challenges of low temperatures and food scarcity during winter. It also provides a means of avoiding natural predators, as hibernating mammals often seek shelter in caves or other concealed locations. Additionally, it potentially extends lifespan, as evidenced by reduced telomere shortening rates in hibernating species such as the Edible Dormouse (*Glis glis*) and Djungarian Hamster (*Phodopus sungorus*) (Turbill et al., 2013; Turbill et al., 2012). Collectively, these adaptations contribute to enhancing the survival rates of hibernating mammals and, from a broader perspective, may contribute to slowing down the extinction rate of species (Geiser and Turbill, 2009).

The intricate composition, intricate structure, and diverse functionality of gut microbiota exert profound influences on the overall health status of their hosts. These microbiota are intricately linked to numerous physiological processes within the host, including metabolism, behavioral patterns, nutritional absorption, and immunological responses (Carey and Assadi-Porter, 2017; Makki et al., 2018; Di Vincenzo et al., 2024). Furthermore, the gut microbiota dynamically adapts its composition and activity in response to the available food resources within the digestive tract and the varying nutritional states of the host, particularly during fasting or hibernation. This comprehensive review examines the specific impacts of hibernation on the gut microbiota and its associated metabolites in animals. It delves into the intricate relationship between the alterations in gut microbiota during hibernation and crucial physiological processes such as host energy metabolism, gut immune function, and nitrogen cycling. By elucidating these interconnections, this paper provides valuable insights and serves as a reference for future investigations into the gut microbiota of hibernating mammals, thus advancing our understanding of the microbiota’s role in the adaptation and survival of animals during periods of inactivity and limited food availability.

## 2 Effects of hibernation on gut microbiota

The combined effects of mammals and gut microbiota have created a complex relationship, which has brought benefits to both sides. The effects of fasting, hypothermia, and low metabolic rate during hibernation on gut microbiota were discussed in the following text.

### 2.1 Effects of fasting (nutritional restriction) on gut microbiota diversity and structure

For hibernators, eating a lot before hibernation and long-term fasting during hibernation, this natural circulating food intake provides an opportunity to check the response of the intestinal microbiome to changes in substrate availability. Hibernation typically leads to a reduction in the diversity of the gut microbiota. During hibernation, the alpha diversity were significantly decreased compared to other periods, which have been found in 13-lined ground squirrel (*Ictidomys tridecemlineatus*), Daurian ground squirrel (*Spermophilus Dauricus*), greater horseshoe bat (*Rhinolophus ferrumequinum*), and brown bear (*Ursus arctos*) (Bao et al., 2023; Yang et al., 2021; Xiao et al., 2019; Sommer et al., 2016). The competition of limited resources in the intestines of hibernators may be an important factor in the formation of microbiota. During hibernation, due to a long-term lack of food intake, only the substrate derived from the host’s intestine (including mucosaccharides, exfoliated epithelial cells, and pancreatic and bile secretions) to support the survival of the intestinal microbiota (Leser et al., 2000; Martens et al., 2008; Backhed et al., 2005). Most host-derived substrates in the mammalian gut are gel-forming mucins, complex glycoproteins produced in large quantities by intestinal goblet cells that can coat the protective mucous layer above the epithelium (McGuckin et al., 2011). In fact, only a few intestinal bacteria have the ability to completely degrade mucin (Derrien et al., 2010; Hoskins et al., 1985; Martens et al., 2008; Salyers et al., 1977), for example, *Akkermansia muciniphila*, which can use mucin as carbon and nitrogen source (Derrien et al., 2010; Derrien et al., 2004). Bacteria that cannot use mucin, such as *Lachnospiraceae* and *Lactobacillus*, which feed on monosaccharides, are almost non-existent during hibernation. Bacteroidetes typically utilize starch, cellulose, and pectin as substrates, but the increase in the relative abundance of Bacteroidetes during hibernation may be due to their ability to metabolize host mucosaccharides, enabling them to metabolize host polysaccharides when dietary polysaccharides are scarce (Sonnenburg et al., 2005).

The gastrointestinal microbiota of hibernating mammals exhibits a distinctive composition, characterized by notable fluctuations in the abundance of specific bacterial groups that are adapted to the physiological conditions associated with torpor. A significant body of research indicates that *Bacteroides*, a genus of bacteria known for their ability to break down complex polysaccharides, consistently dominates the intestinal ecosystem of various hibernating species such as the 13-lined ground squirrel (*Ictidomys tridecemlineatus*), Arctic ground squirrel (*Spermophilus parryii*), and brown bear (*Ursus arctos*) during their period of inactivity (Dill-Mcfarland et al., 2014; Stevenson et al., 2014; Sommer et al., 2016). However, these general trends are subject to exceptions reflective of the complex adaptive strategies employed by different microbial communities. In particular, studies have shown a reduced representation of the *Prevotella* within the gut microbiota of 13-lined ground squirrels and Arctic ground squirrels during hibernation, contrasted against non-hibernating periods (Dill-Mcfarland et al., 2014; Stevenson et al., 2014). This observation is attributed to the limited capacity of *Prevotella* spp. to degrade host-derived mucous polysaccharides, which become the predominant substrates in the absence of dietary intake (Ley, 2016). Conversely, the family *Enterobacteriaceae*, known for its capacity to metabolize intestinal mucopolysaccharides, has been observed to increase in relative abundance in hibernating brown bears (Ma et al., 2012). The restructuring of the gut microbiota during hibernation appears to be driven by substrate preferences of the constituent microorganisms. Those capable of utilizing host-derived substrates, such as members of the *Verrucomicrobia* and certain taxa within the Bacteroidetes phylum, may undergo proliferation due to their ability to modulate carbohydrate-degrading enzymes in response to variations in nutrient availability (Sonnenburg et al., 2005). Concomitantly, there is often a depletion of microbial species that rely on dietary polysaccharides, including many within the Firmicutes phylum, suggesting a selective pressure favoring those adapted to metabolic host-derived compounds over those specialized in plant polysaccharide degradation. Table 1 summarized the changes in gut microbiota during mammalian hibernation.

**Table 1:** Changes in major microbial communities during hibernation.

Phylum		Microbiota	Reason	Mammals	Reference
Firmicutes	Increased	*Rumino_x0002_coccaceae UCG-014* *Coprococcus* *Aerococcaceae*	Produce butyric acid to maintain gut barrier Adapt to lower intestinal pH	13-lined ground squirrels Arctic ground squirrels Brown bear Siberian Chipmunk Dwarf lemurs Himalayan marmot Greater Horseshoe Bat	Dill-Mcfarland et al. (2014), Bao et al. (2023), Xiao et al. (2019), Sommer et al. (2016), Zhou et al. (2022), and Stevenson et al. (2014)
	Decreased	*Lachnospiracea* *Anaerotruncus* *Lactobacillaceae* *Streptococcaceae*	Plant polysaccharides as substrates Carbohydrates as substrates Lack the ability to degrade host mucus polysaccharides		
Bacteroidetes	Increased	*Porphyromonadaceae* *Rikenellaceae* *Bacteroides*	Host mucus polysaccharides as substrates	13-lined ground squirrels Arctic ground squirrels Himalayan marmot Greater Horseshoe Bat	Dill-Mcfarland et al. (2014), Stevenson et al. (2014), Sommer et al. (2016), Bao et al. (2023), Xiao et al. (2019), and Sonnenburg et al. (2005)
	Decreased	*Prevotella*	Lack the ability to degrade host mucus polysaccharides		
Verrucomicrobia	Increased	*Verrucomicrobiaceae* *Akkermansia muciniphila*	Host gastrointestinal mucin protein as substrate	Greater Horseshoe Bat Syrian hamster 13-lined ground squirrels Arctic ground squirrels	Xiao et al. (2019), Sonoyama et al. (2009), Dill-Mcfarland et al. (2014), Stevenson et al. (2014), Sonnenburg et al. (2005), Derrien et al. (2010), and Derrien et al. (2004)
Actinobacteria	Decreased	*Coriobacteriaceae* *Micrococcus*	Lack the ability to degrade host mucus polysaccharides	Brown bear 13-lined ground squirrels	Sommer et al. (2016) and Dill-Mcfarland et al. (2014)
Proteobacteria	Increased	*Pseudomonas* *Enterobacteriaceae*	Wide metabolic diversity, including gastrointestinal mucopolysaccharides	13-lined ground squirrels Greater Horseshoe BatBrown bear	Dill-Mcfarland et al. (2014), Xiao et al. (2019), and Ma et al. (2012)

However, hibernation patterns also vary among different hibernators. Depending on the specific strategies they employ to acquire energy during their inactive period, hibernating mammals can be categorized into two groups: fat-storing hibernators and food-storing hibernators. Fat-storing hibernators accumulate energy-rich substances primarily in the form of body fat to sustain them through the winter, whereas food-storing hibernators stockpile a significant quantity of non-decaying seeds before entering hibernation to ensure a constant source of nourishment (Humphries et al., 2003). Remarkably, despite their different energetic approaches, the gut microbiota of both fat-storing and food-storing hibernators share notable similarities (Xiao et al., 2019). Food-storing hibernators, such as hamsters, exhibit minimal changes in their microbiota composition during hibernation, suggesting a stability that is maintained even under conditions of reduced food intake. However, when these animals are subjected to fasting conditions similar to those experienced by ground squirrels (for instance, a four-day fast), they display microbiota alterations that are comparable to those observed in the latter species (Sonoyama et al., 2009). Collectively, these studies provide compelling evidence that a core intestinal microbiota may exist during hibernation in mammals. This core microbiota appears to be resilient and capable of maintaining functional stability across diverse hibernation strategies, highlighting the adaptability and resilience of the gut microbiome in response to the physiological challenges of hibernation.

However, these studies offer an incomplete understanding of the intricate relationship between hibernation, dietary habits, and the microbiome, considering that squirrels, for instance, maintain a minimum body temperature that approaches or slightly dips below 0°C during hibernation. Consequently, the specific factors that drive microbiota differences during hibernation, whether dietary intake, hypothermic conditions, or a combination of both, remain elusive. Until 2016, Sommer et al. made a significant discovery in brown bears, a large hibernating mammal. They observed a notable reduction in gut microbiota diversity during hibernation, accompanied by a decrease in the relative abundance of Firmicutes and an increase in Bacteroidetes (Sommer et al., 2016). Notably, brown bears do not experience a drastic drop in body temperature during hibernation, suggesting that winter fasting in these animals primarily accounts for the depletion of degradable substrates that typically support gut microbial growth. This deprivation may significantly influence the structure of the microbial community and its interactions with the host. Consistent with these findings, similar patterns have been observed in studies focusing solely on fasting. For instance, the proportion of *A. maciniphila* within the microbiota of fasting hamsters increased, while the proportion of Firmicutes decreased (Sonoyama et al., 2009). Similarly, mice subjected to a 24-h fasting period exhibited an increase in Bacteroidetes and a decrease in Firmicutes in their intestinal tract (Crawford et al., 2009). These results collectively indicate that fasting during hibernation is a critical factor that significantly shapes the composition and proportion of the gut microbiota.

### 2.2 Effects of low temperature on gut microbiota

In addition to fasting, hypothermia—characterized by a significant drop in body temperature—emerges as a pivotal factor that shapes the composition and structure of the gut microbiota. Animals, in their natural habitats, must adapt to fluctuations in food availability and temperature extremes, resulting in dynamic changes in the species and proportions of gut microorganisms (Stevenson et al., 2014). This adaptation process is particularly evident in wildlife species residing in diverse environments. For instance, the plateau pika (*Ochotona curzoniae*), a species that inhabits alpine regions with harsh climatic conditions, exhibits a higher diversity of gut microorganisms compared to its low-altitude counterpart, the Daurian pika (*Ochotona daurica*) (Li et al., 2018). This observation suggests that cold environments can significantly impact the composition of the gut microbiota. Similarly, studies on Brandt’s voles (*Lasiopodomys brandtii*) have revealed that exposure to low temperatures modifies the composition and proportions of gut bacteria, such as an increase in *Ruminococcus* and *Helicobacter* species. While these studies primarily focus on ambient temperature, it is noteworthy that for homeothermic animals, variations in body temperature serve as the direct influencing factors on the gut microbiota. Dill-Mcfarland et al. conducted research demonstrating that as body temperature decreases in homeothermic animals, there are consequent changes in the gut microbiota. These include a decrease in bacterial diversity, a reduction in the relative abundance of Firmicutes, and a decrease in pathogenic bacteria like *Salmonella*. Concurrently, there is an increase in the number of Bacteroidetes (Dill-Mcfarland et al., 2016). Therefore, during hibernation, where animals experience both limited food resources and hypothermic conditions, both factors play crucial roles in determining the composition and structure of the gut microbiota. Hibernation, thus, represents a unique physiological state that significantly alters the gut microbial ecosystem, necessitating further investigation to fully understand its complex interactions and potential consequences for animal health.

### 2.3 Effects of hypometabolism on gut microbiota

Hibernation, an energy-conserving survival strategy utilized by numerous mammalian species, is characterized by a significant reduction in metabolic rate (Storey and Storey, 2004). This reduction, which can plummet to merely 4.3% of the animal’s basal metabolic rate (Ruf and Geiser, 2015), is pivotal in maintaining energy homeostasis during periods of limited food availability. During hibernation, the gut microbiota of hibernating mammals assumes a crucial role in maintaining the health and function of the intestinal tract by feeding on intestinal substrates. Recent studies, such as the one conducted by Bo et al., have revealed that modifications in the gut microbiota composition of hibernating mammals, such as Brandt’s voles, are significantly associated with the host’s resting metabolic rate (RMR) after undergoing low-temperature domestication or microbial transplantation. Similarly, in laboratory mice, alterations in the gut microbiota have been intimately linked to both RMR and nonshivering thermogenesis (NST). These findings suggest that the gut microbiota plays a pivotal role in regulating metabolic processes in hibernating mammals. Among the various microbial species present in the gut, *Desulfovibrio* has been identified as a key player. Earley et al. (2015) demonstrated that *Desulfovibrio* can metabolize sulfates derived from mucin, ultimately producing hydrogen sulfide (H2S). Notably, H2S, as a potent inhibitor of cytochrome C oxidase, is implicated in metabolic suppression during hibernation (Nicholls et al., 2013; Revsbech et al., 2014). While the exact mechanisms underlying the interaction between hibernation, metabolic suppression, and gut microbiota remain incompletely understood, it is reasonable to speculate that the low metabolism observed during hibernation is among the factors that shape and influence the composition and activity of the gut microbiota.

## 3 Effects of hibernation on gut microbiota metabolites

The genetic diversity of gut microbiota during hibernation endows this intricate system with an array of enzymes and biochemical metabolic pathways that are absent in the host. This diversity enables the microbiota to harness energy through the fermentation and catabolism of polysaccharides (including starch, cellulose, and pectin) as well as unabsorbed oligosaccharides. The breakdown of these diverse substrates results in the production of distinct metabolites, each exerting unique physiological effects on the host. Among the metabolites generated by the gut microbiota, short-chain fatty acids (SCFAs) such as acetic acid, propionic acid, butyric acid, and valeric acid stand out as particularly significant. Once absorbed by the host, these SCFAs serve as a source of energy (McNeil, 1984; Koh et al., 2016). Specifically, butyric acid fulfills the energetic demands of intestinal epithelial cells and ensures the proper growth of intestinal mucosal cells. Meanwhile, propionic acid contribute to the host’s glucose metabolism (Venter et al., 1990). When comparing intestinal SCFAs concentrations between hibernation and summer periods (assuming similar body temperatures), a decrease of approximately 30% is observed during hibernation. SCFA levels reach their nadir during the hibernation phase but subsequently rise during arousal, potentially attributed to the stimulatory effect of elevated body temperature on bacterial enzyme activity. Additionally, hibernation induces alterations in the molar ratio of individual SCFAs (Carey et al., 2013). During hibernation, the gut microbiota undergoes spontaneous structural reorganization, modifying its composition. Under conditions of prolonged fasting, these microorganisms maintain a balanced relationship with their hosts, often characterized as mutualistic. This is evident as intestinal epithelial cells derive 60–70% of their energy requirements from SCFAs produced by microbial metabolism (Ardawi and Newsholme, 1985). Conversely, the epithelial cells provide a favorable niche for the hibernating microbiota to persist by offering components such as epithelial cells and mucin glycans for their survival (Carey and Martin, 1996). Notably, the impact of hibernation on individual SCFAs varies. Acetate maintains the highest molar ratio throughout all seasons, with its proportion further increasing during hibernation (Stevenson et al., 2014). These changes in metabolites are summarized in Table 2, highlighting the dynamic interplay between hibernation, gut microbiota, and their metabolic outputs.

**Table 2:** Changes in gut microbiota metabolites during hibernation.

Metabolite	Microbiota	Change	Function
Acetate	*Akkermansia muciniphila* *Bifidobacterium* spp. *Ruminococcus* spp.	Increased	Energy source for intestinal epithelial cells Ketogenesis in the liver, providing energy to the brain and heart
Butyrate	Firmicutes *Roseburia Eubacterium Faecaliberium*	Decreased	Stimulates epithelial cell proliferation and recovery, and aids in maintaining the integrity of the intestinal barrier by regulating apoptosis, permeability, and mucus production
Propionate	*Bacteroides* spp. *Dialister* spp. *Coprococcus catus*	Unchanged	Substrate for gluconeogenesis, another metabolic function crucial for maintaining blood glucose levels during hibernation
Lactic acid	*Lactobacillus* *Lactococcus*	Decreased	Source of energy and regulate lipid and carbohydrate metabolism in aerobic environments
Succinic acid	*Enterobacteriaceae*	Increased	Succinic acid is an intermediate substance in the tricarboxylic acid cycle and plays an important role in ATP generation

Acetate: During hibernation, the proportion of acetate rises, partially due to an increase in *Akkermansia muciniphila*. Microbially-derived acetate can affect hibernation physiology in multiple ways. Intestinal epithelial cells and peripheral gut tissues can utilize acetate as an energy source, and acetate transported to the liver can also be used for the synthesis of fatty acids and cholesterol (Macfarlane and Macfarlane, 2003). Acetate contributes to ketogenesis in the liver (Crawford et al., 2009), which can serve as an alternative fuel to glucose, providing energy to the brain and heart (Andrews et al., 2009), protecting the heart from ischemia–reperfusion injury, and facilitating the transition of animals from a hypometabolic to a hypermetabolic state upon arousal from hibernation.

Butyrate: During hibernation, butyrate levels in the host’s gut decrease to very low levels. This may be attributed to the reduction of certain types of metabolizable substrates and alterations in the composition of the microbiota. The lack of complex plant polysaccharides during hibernation diminishes the Firmicutes phylum, subsequently reducing butyrate production, as most major butyrate producers, including Roseburia, Eubacterium, and Faecaliberium, belong to the Firmicutes (Duncan et al., 2007). The lower cecal butyrate concentration in winter may contribute to changes in the gut structure and function of hibernating squirrels. As the preferred fuel source for epithelial cells, butyrate stimulates epithelial cell proliferation and recovery, and aids in maintaining the integrity of the intestinal barrier by regulating apoptosis, permeability, and mucus production (Louis and Flint, 2009; Peng et al., 2007; Brahe et al., 2013).

Propionate: In hibernating mammals, the proportion of propionate remains unchanged (12–13%) (Stevenson et al., 2014). Propionat produced by microbial metabolism, is a substrate for gluconeogenesis, another metabolic function crucial for maintaining blood glucose levels during hibernation (Galster and Morrison, 1975).

Lactic acid and succinic acid: During animal hibernation, bacteria in the gut microbiota that produce lactic acid, such as *Lactobacillus* and *Lactococcus* (Wells and Mercenier, 2008), decrease in their relative abundance, leading to a decrease in lactate levels in the intestine. L-Lactate can serve as an important source of energy and regulate lipid and carbohydrate metabolism in aerobic environments. In hibernating brown bears, the relative abundance of *Enterobacteriaceae* significantly increases compared to non hibernating periods. The increase in succinic acid content during hibernation is related to the relative abundance of succinic acid producing bacteria such as *Enterobacteriaceae* (Sommer et al., 2016). Succinic acid is an intermediate substance in the tricarboxylic acid cycle and plays an important role in ATP generation (Mills and O’Neill, 2014).

## 4 Effects of gut microbiota on the host during hibernation

### 4.1 Gut microbiota affects energy metabolism

**Figure 1:**
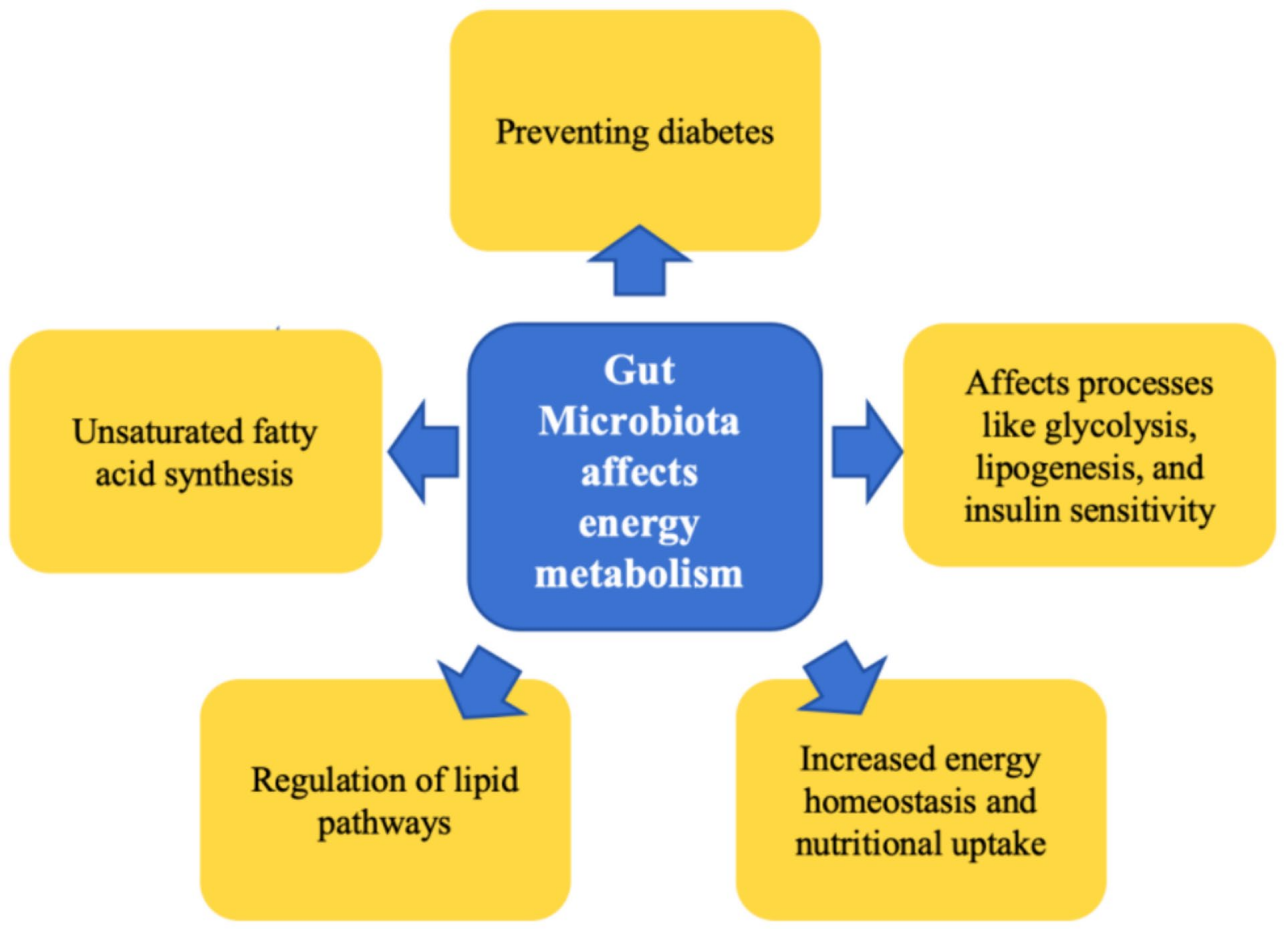
Summary of the role of gut microbiota in energy metabolism during hibernation.

The resident gut microbiota significantly influences the host’s nutritional uptake and energy homeostasis by augmenting the extraction of energy from ingested food and facilitating its storage within the body (Bäckhed et al., 2004; Crawford et al., 2009). This microbial community contributes substantially to the maintenance of energy metabolic phenotypes in hibernating mammals. Through fecal microbiota transplantation experiments using brown bears, it has been discovered that the microbial communities native to these animals can modulate recipient mice phenotypes, resulting in increased body weight, obesity, and insulin resistance (Sommer et al., 2016). The gut microbiota, along with its metabolites such as short-chain fatty acids (SCFAs), play an integral role in maintaining equilibrium within the host’s glucose and lipid metabolism, affecting processes like glycolysis, lipogenesis, and insulin sensitivity (Zhang et al., 2023; Wang et al., 2023; Anhê et al., 2023). Research findings from Bao et al. (2023) suggest that Himalayan marmots exhibit a dietary preference for unsaturated fatty acids (UFA) prior to hibernation, hypothesized to facilitate fat accretion in these animals. Concurrently, the class Firmicutes, specifically the cluster CAG: 110, partakes in UFA synthesis, thus aiding in supporting the host’s metabolic processes during hibernation. In a subsequent fecal transplantation experiment, there was an observed increment in the weight of the recipient mice, further corroborating the upregulating effect of Firmicutes CAG: 110 on lipid metabolism during the hibernation phase. In hibernaors that store fat, specific lipid types fulfill distinct functional roles in both regular physiology and during hibernation. Lipids such as cholesterol and fatty acids can regulate pathways associated with torpor. For instance, dietary cholesterol enrichment in chipmunks enhances hibernation parameters (Kolomiytseva, 2011), and provisioning Alpine marmots with an omega-6 fatty acid-rich diet can ameliorate survival rates during hibernation (Ruf and Arnold, 2008). Studies on bears also reveal that a specialized lipid metabolic mode is instrumental in preventing diabetes during hibernation (Tekin et al., 2023). Therefore, during the hibernation period, the gut microbiota undergo transformations due to fasting and reduced temperatures. Such changes are instrumental in sustaining the energy metabolic phenotype throughout the hibernation cycle (Figure 1).

### 4.2 Gut microbiota affects immunity

During hibernation, fasting, low temperature conditions, and alterations in gut microbiota converge to induce significant remodeling of the host’s intestinal immune system. A depletion of SCFAs is hypothesized to be the primary culprit behind the atrophy of the small intestinal mucosa and the subsequent elevation in intestinal barrier permeability, as described by Carey (1992). This increase in permeability poses a greater risk for bacterial translocation and the hyperactivation of the immune system. Butyrate, being the primary energy source for epithelial cells, its absence results in suppressed proliferation of these cells, decreased integrity of the intestinal barrier, and consequently, elevated intestinal permeability (Gaudier et al., 2004; Louis and Flint, 2009; Bugaut and Bentéjac, 1993). Nonetheless, the gut microbiota plays a pivotal role in maintaining immunity during hibernation. Notably, during hibernation, there is a reduction in the number of microorganisms within the intestinal lumen, coupled with an increase in those adherent to the mucosal layer. The proximity of these mucosal microorganisms to the intestinal epithelium allows for direct interactions with host cells and indirect regulation of the immune system through the diffusion of microbial-associated molecular patterns (MAMPs) or their metabolites (Van den Abbeele et al., 2011). Research on the 13-lined ground squirrel demonstrates that hibernation is associated with shortened caecal crypts and enhanced MUC2 expression during early winter and spring compared to summer. Additionally, hibernation is observed to decrease the expression of TLR4 and increase TLR5 in the caecum, suggestive of a protective response aimed at minimizing inflammation. Activation of TLR5 by its ligand, flagellin, elicits a protective anti-inflammatory response and maintains intestinal barrier function (Cullender et al., 2013). Therefore, the intricate interaction between toll-like receptor expression in epithelial cells and the mucosal microbiota appears to constitute a protective mechanism for intestinal immunity during hibernation (Carey et al., 2013). This enhancement of mucosal immunity serves to minimize inflammation triggered by microbial leakage from the intestine, thereby safeguarding intestinal health (Maynard et al., 2012).

Alterations in the immunological status are frequently correlated with the detection of host-derived microbial signals, which may contribute to the establishment and maintenance of a mutualistic and tolerogenic equilibrium (Maynard et al., 2012). The period of hibernation is characterized by shifts in the gut microbiota and their metabolic byproducts, which can elicit an augmentation in the population of intraepithelial and lamina proprialymphocytes, augmented expression of immunoglobulin A (IgA), and heightened concentrations of mucosal cytokines (Kurtz and Carey, 2007; Hooper et al., 2012). The role of IgA as a pivotal regulatory element in the intricate relationship between the commensal microbiota, epithelial cells, and the immune system is well established (Peterson et al., 2007; Sutherland and Fagarasan, 2012). The hibernation phase also induces an enhanced distribution of occludin within the intestinal and caecal epithelia, a tight junction protein instrumental in fortifying the intestinal barrier, particularly under conditions of inflammation (Carey et al., 2012; Turner, 2009). Concurrently, the hibernation state is associated with an upregulation of mucosal interleukin-10 (IL-10), an efficacious anti-inflammatory cytokine that fosters immunological tolerance toward the resident microbial community (Flint et al., 2012; Hooper et al., 2002). Furthermore, the intestinal epithelium’s defensive mechanisms in hibernating species are reinforced through an increased expression of proteins that counteract apoptosis (Dill-McFarland et al., 2014).

The intestinal microbiota performs a pivotal function in immunity. For instance, *Bacteroides thetaiotaomicron* demonstrates the ability to degrade host glycans, even in the absence of dietary polysaccharides. This bacterium not only assists the host in efficiently utilizing nutrients, such as carbohydrates, but it also enhances immune responses and contributes significantly to maintaining the delicate balance of the intestinal microecosystem (Flint et al., 2012; Koropatkin et al., 2012). During hibernation, a noteworthy shift occurs in the intestinal microbiota of brown bears, specifically with an increase in the proportion of *Coprococcus* species. This bacterium is capable of producing butyrate, a compound that is essential for preserving the integrity and function of the intestinal barrier. Additionally, *Akkermansia muciniphila* stimulates the production of mucus by the host, thereby strengthening the integrity of epithelial cells (Derrien et al., 2004; Geerlings et al., 2018). This bacterial species further enhances intestinal immune function during hibernation, ensuring that the bear maintains a robust immune defense despite its inactive state.

### 4.3 Gut microbiota affects urea metabolism

The urea nitrogen cycle plays a pivotal role in maintaining protein homeostasis and averting ammonia toxicity in animals during hibernation (Lee et al., 2012; Rice et al., 2020). Mammalian hibernators employ sophisticated mechanisms to minimize nitrogen losses during prolonged winter fasting, thereby preserving vital nutrients (Nelson, 1989). Within the intestine, the gut microbiota facilitates the hydrolysis of urea into carbon dioxide and ammonia. Subsequently, this ammonia can be further metabolized into amino acids, which contribute to the biosynthesis of new proteins (Carey and Assadi-Porter, 2017; Regan et al., 2022). This microbial “recycling” of urea nitrogen back to the host represents a crucial aspect of host-microbiota cometabolism, significantly contributing to the metabolic phenotype of hibernating mammals. In ground squirrel species, the downregulation of urea cycle enzymes during hibernation suggests a preferential utilization of bacterial-derived urea nitrogen for amino acid synthesis rather than urea production (Epperson et al., 2010). Consequently, during winter hibernation, both the plasma urea concentration and intestinal NH3 levels are significantly reduced in ground squirrels compared to their summer state (Regan et al., 2022). Notably, the enhanced nitrogen metabolism observed during hibernation exhibits a negative correlation with various physiological parameters such as body weight, food intake, blood glucose, and body temperature in Daurian ground squirrels. This suggests that the cecal microbiota of these animals plays a vital role in converting potential toxic substances through nitrogen metabolism, thereby providing supplemental energy to the host during hibernation. Regan et al.’s study further highlights the ability of ground squirrels to harness their gut microbiota for urea nitrogen recycling, ensuring protein balance even in the absence of dietary nitrogen sources (Regan et al., 2022). This mechanism confers two key benefits to hibernating mammals: Firstly, it enhances protein synthesis during periods of nitrogen scarcity, crucial for ground squirrels approaching the breeding season during the later stages of hibernation, potentially translating into reproductive advantages. Secondly, the urea nitrogen cycle redirects urea away from the kidneys, reducing the water requirements for urine production and thus conserving precious bodily resources for hibernating mammals.

## 5 Significance of hibernation microbiota research

The intricate co-evolutionary between mammals and their gut microbiota has engendered a highly complex and mutually beneficial relationship. This symbiosis is characterized by the changes in microbial structure, modulation of intestinal structure and function, stimulation of immune system development, and enhancement of dietary energy extraction. At the same time, the host mammal provides an array of nutritional and environmental niches tailored to the microbiota’s needs, while also shaping its composition and structure through genetic predisposition, immune status, intestinal milieu, and dietary patterns.

The exploration of the gut microbiota in hibernating mammals holds particular scientific significance due to several key reasons. Firstly, hibernating mammals present a unique and naturally occurring model system for elucidating the intricate relationship between gut microbiota and host co-evolution. Secondly, investigating the dynamic shifts in gut microbiota composition during hibernation cycles offers insights into the microbiota’s regenerative capabilities and its impact on hibernators’ rapid transition into reproductive readiness following arousal. Thirdly, the study of gut microbiota in hibernators and their urea nitrogen salvage mechanisms reveals a functional role for intestinal microorganisms in this adaptive physiological process, providing valuable knowledge about the microbiota’s role in metabolic regulation. Lastly, understanding the adaptive mechanisms employed by hibernators and their gut microbiota to cope with nutritional deprivation during winter fasting can inform our comprehension of potential protective mechanisms against long-term fasting in non-hibernating species, such as humans. This knowledge has the potential to inform nutritional strategies, therapeutic interventions, and even inform the design of novel microbiome-based therapeutics.
